# Gender Differences in HIV Care among Criminal Justice-Involved Persons: Baseline Data from the CARE+ Corrections Study

**DOI:** 10.1371/journal.pone.0169078

**Published:** 2017-01-12

**Authors:** Curt Beckwith, Breana Uhrig Castonguay, Claudia Trezza, Lauri Bazerman, Rudy Patrick, Alice Cates, Halli Olsen, Ann Kurth, Tao Liu, James Peterson, Irene Kuo

**Affiliations:** 1 The Miriam Hospital, Providence, RI, United States of America; 2 Brown University Alpert School of Medicine, Providence, RI, United States of America; 3 George Washington University Milken Institute School of Public Health, Washington, DC, United States of America; 4 Yale University School of Nursing, New Haven, CT, United States of America; 5 Brown University School of Public Health, Providence, RI, United States of America; University of New South Wales, AUSTRALIA

## Abstract

**Background:**

HIV-infected individuals recently released from incarceration have suboptimal linkage and engagement in community HIV care. We conducted a study to evaluate an information and communication technology intervention to increase linkage to community care among HIV-infected persons recently involved in the criminal justice (CJ) system. Baseline characteristics including risk behaviors and HIV care indicators are reported and stratified by gender.

**Methods:**

We recruited HIV-infected individuals in the District of Columbia jail and persons with a recent history of incarceration through community and street outreach. Participants completed a baseline computer-assisted personal interview regarding HIV care and antiretroviral treatment (ART) adherence, substance use, and sexual behaviors. CD4 and HIV plasma viral load testing were performed at baseline or obtained through medical records. Data were analyzed for the sample overall and stratified by gender.

**Results:**

Of 110 individuals, 70% were community-enrolled, mean age was 40 (SD = 10.5), 85% were Black, and 58% were male, 24% female, and 18% transgender women. Nearly half (47%) had condomless sex in the three months prior to incarceration. Although drug dependence and hazardous alcohol use were highly prevalent overall, transgender women were more likely to have participated in drug treatment than men and women (90%, 61%, and 50% respectively; p = 0.01). Prior to their most recent incarceration, 80% had an HIV provider and 91% had ever taken ART. Among those, only 51% reported ≥90% ART adherence. Fewer women (67%) had received HIV medications during their last incarceration compared to men (96%) and transgender women (95%; p = 0.001). Although neither was statistically significant, transgender women and men had higher proportions of baseline HIV viral suppression compared to women (80%, 69%, and 48.0% respectively, p>0.05); a higher proportion of women had a CD4 count ≤200 compared to men and transgender women (17%, 8% and 5% respectively; p>0.05).

**Conclusions:**

In this study, HIV-infected persons with recent incarceration in Washington, DC reported important risk factors and co-morbidities, yet the majority had access to HIV care and ART prior to, during, and after incarceration. Self-reported ART adherence was sub-optimal, and while there were not statistically significant differences, CJ-involved women appeared to be at greatest risk of poor HIV outcomes.

**Trial registration:**

Registered on ClinicalTrials.gov on 10/16/2012. Reference number: NCT01721226.

## Background

Criminal justice (CJ)-involved persons, defined as persons with a history of incarceration in prison or jail and persons who have been under community corrections supervision (probation, parole), are disproportionately impacted by HIV infection. A high prevalence of substance use disorders, mental health disorders and racial and ethnic health disparities contribute to this increased burden [1, 2]. CJ populations experience high rates of unstable housing, unemployment, and poverty, which negatively influence retention in longitudinal HIV care [3]. Incarceration and subsequent release from a correctional facility have been associated with interruptions in antiretroviral treatment (ART) [4], loss of viral control [5] and failure to maintain adherence to ART [4, 6, 7]. Approximately one in seven HIV-infected persons in the United States (U.S.) passes through the CJ system annually [8], providing an opportunity to address the HIV care continuum [3] and to deliver interventions designed to improve access to HIV care and ART.

The majority of persons involved in the U.S. criminal justice system are men, with women representing only 7% of the prison population and up to 25% of persons on probation in the community [9, 10]. CJ-involved women and transgender persons have unique health challenges, yet these populations remain understudied. Incarcerated women have higher rates of substance use and addiction [11, 12], HIV infection [13, 14],hepatitis C virus infection [15], and mental health disorders [16, 17] compared to incarcerated males [18]. Transgender persons, particularly male-to-female (MTF) transgender women, experience high rates of HIV infection [19, 20], clinical depression [21], unstable housing [22], and gender violence and economic discrimination [23], which can contribute to risky sexual behaviors [24, 25].

Persons who cycle in and out of jails and prisons are at risk of transmitting HIV to sexual and drug-using partners; therefore, developing and implementing interventions to improve the delivery of HIV treatment to CJ-involved persons in order to achieve viral suppression is a public health priority. The Seek, Test, Treat, and Retain (STTR) strategy to control the HIV epidemic has been proposed as a public health approach to improve HIV outcomes through greater access to ART [26–28]. The STTR strategy is multi-pronged and includes increasing the identification of HIV-infected persons through expanded testing, linkage to HIV providers, improving access and adherence to ART, and maintaining long-term retention in care. If achieved, this, in turn, decreases community transmission of HIV by creating a population of HIV-infected persons who are less infectious by effective ART [29].

In 2010, the National Institutes of Health/National Institute on Drug Abuse funded a research initiative to investigate the application of the STTR strategy among CJ-involved persons [25]. As part of this initiative, we launched the CARE+ Corrections Study conducted in Washington, D.C., a city with an HIV prevalence estimated to be at 2.5% of all residents and 5.8% among Black males [30]. The goal of the parent study was to develop and evaluate the efficacy of a technology-based intervention designed to improve linkage to community HIV providers and ART adherence following release from a correctional facility. With this intervention, our study aimed to address the Treat and Retain aspects of the STTR strategy using an intervention to leverage information and communication technology (ICT) tools. ICTs have the potential to be scalable among correctional facilities and community partners who assist persons released from prison or jail, also referred to as “returning citizens”. Given the gender diversity of the CARE+ Corrections Study participants, we herein describe their baseline characteristics and compare risk behaviors and HIV care indicators across men, women, and transgender persons.

## Methods

### Study Design

The CARE+ Corrections Study was originally designed as a randomized, controlled, longitudinal trial to evaluate the efficacy of the CARE+ Corrections intervention among a total study population of 320 participants. However, we encountered challenges with study implementation and participant enrollment within the Washington, DC Department of Corrections (DC DOC) facilities and were therefore unable to enroll a sufficient number of participants to evaluate the efficacy of the intervention. The study objective was changed to be a preliminary evaluation of feasibility and efficacy among 100 study participants, and enrollment was adapted to include persons recruited inside the correctional facilities and persons in the community recently released from a correctional facility, thus providing a convenience sample of persons involved with the criminal justice system. Recruitment within correctional facilities was stratified to ensure enrollment of at least 25% females; recruitment in the community was not stratified based upon gender. Within strata, participants were randomly assigned to either the CARE+ Corrections intervention or the control arm on a 1:1 basis using a computer-generated randomization scheme.

### Intervention and Control Groups

The CARE+ Corrections Study intervention contained two components. The first was a computerized counseling session (“CARE+ Corrections”). This was an adapted version of the CARE+ counseling tool, a one-session, interactive, computerized motivational interview that assessed a person’s risk and HIV care behaviors (including HIV care utilization, ART adherence, and retention in HIV care) and provided an individualized risk reduction plan for ART adherence or linkage to care [31, 32, 33]. CARE+ Corrections was specifically designed to provide counseling to HIV-infected persons being released from correctional facilities. Counseling content focused on linkage to community HIV care, ART adherence, and reducing risk behaviors [31, 34]. Participants received a printout at the end of the session that summarized goals and provided relevant HIV treatment referrals.

The second component of the CARE+ Corrections Study intervention was scheduled text messages to the participant following release from a correctional facility. The text messages included supportive behavioral messaging (e.g., “One day at a time. Just for today, don't use.”) as well as medication and appointment reminder messages. Participants were able to select pre-scripted messages or create their own messages, and to select frequency and timing of messaging (e.g., daily, weekly, morning, evening). All participants in the intervention group received a cell phone and mobile service supported by the study in order to ensure the receipt of study-based text messages.

Participants in the control group watched an educational video on opioid overdose prevention and received a printout of local HIV providers and resources. The length of the video was approximately equal to the time intervention group participants spent completing the CARE+ Corrections counseling session. Participants in the control group did not receive a cell phone or the text messaging intervention.

### Study Population and Eligibility

The target population was HIV-infected individuals with a recent history of incarceration. Study eligibility included being at least 18 years old, HIV-infected by self-report, incarcerated in the DC DOC with an anticipated release date within six weeks or residing in the community but having been released from a jail, prison or halfway house within the previous six months (confirmed by study staff using public records), planning on remaining in the geographic area through the end of the study period, and able to provide informed consent for study participation.

### Recruitment

Recruitment occurred in DC DOC facilities and in the community. Within DC DOC facilities, participants were recruited in either the Central Detention Facility, which houses men and male-to-female transgender persons, or the Correctional Treatment Facility, which houses men and women and includes a 100-bed treatment facility for persons with substance use disorders. The screening and recruitment occurred in the medical units and treatment facility of the DC DOC where HIV care is provided by a contracted medical provider. In these locations, study staff provided information about the study to potentially interested participants, conducted eligibility assessments, and obtained written informed consent and enrolled participants. HIV-infected persons incarcerated within the DC DOC received comprehensive HIV care including access to ART. Discharge planning services at the time of release included provision of a 30-day supply of HIV medications and a referral to community HIV providers.

Individuals from the community were recruited through a combination of street-based recruitment, advertisement at and referrals from local community-based organizations that provide services to returning citizens (such as the Mayor’s Office of Returning Citizens Affairs, shelters, transitional housing programs, female and transgender-friendly agencies, and drug treatment programs), and referrals from other study participants. Persons interested in the study were scheduled for eligibility screening, consenting and completion of the baseline visit at a research clinic in the community. Participants were enrolled between August 24, 2013 and April 30, 2015, with participant follow-up continuing through December 10, 2015.

### Data Collection

Although enrolled participants had follow-up assessments at 3 months and 6 months, we report only on data collected at the baseline visit. The baseline visit included an interviewer-administered structured assessment covering domains related to demographic characteristics, criminal justice history, HIV care engagement, medication adherence, sexual and substance use behaviors, and mental health and other co-morbid conditions. Participants provided a whole blood specimen obtained by venipuncture to measure the baseline HIV plasma viral load (PVL) and CD4 cell count; and completed an in-depth locator form for study retention purposes. Participants enrolled within DC DOC facilities completed the baseline appointment over two visits so that only non-sensitive behavioral data (e.g., demographics, health care utilization and medication adherence prior to current incarceration) were collected inside of the facility. Following release, these participants were scheduled within one week of release to complete the baseline visit (including assessment of sexual and substance use behaviors and venipuncture for a blood specimen to conduct the HIV PVL and CD4 count). Specimens were sent to Miriam Hospital in Rhode Island for HIV PVL testing conducted by the Roche Cobas AmpliPrep/ Cobas Taqman HIV-1 Test, Version 2.0. CD4 cell count testing was completed by LabCorp via the Becton Dickinson Canto II flow cytometer. When study staff were unable to obtain blood from venipuncture or when a DOC-recruited participant did not return to complete the baseline visit in the community following release, DOC or community medical records from previous the six month period were abstracted to collect HIV PVL and CD4 count data to establish baseline values. Informed consent for medical records abstraction was obtained at the time of study enrollment.

### Variable Definitions

Gender was defined based on a series of questions about gender identity and sex assignment at birth. For the purposes of this analysis, a participant was designated as transgender if the individual reported a “transgender” gender identity or if their reported gender differed from their reported sex assignment at birth. Criminal justice experience included the length of the most recent incarceration and the number of times ever been in jail, prison, or juvenile detention. Sexual risk behaviors in the three months prior to their most recent incarceration were assessed, including condomless sex, exchange sex, number of sexual partners, and if designated as male or as a transgender woman, ever and recent sex with a male partner. Substance use was assessed for alcohol and both injection and non-injection drugs during the three months prior to incarceration, as well as lifetime use of injection drugs. Drug dependence, categorized as “not drug dependent” and “drug dependent”, was assessed using the TCU Drug Dependence scale and hazardous alcohol use was determined using the WHO-AUDIT with participants categorized as low, medium or high hazardous harmful alcohol use, both of which have been previously validated [35, 36]. Symptoms related to post-traumatic stress disorder (PTSD) and depression were assessed using the Primary Care PTSD Screen and the CES-D instruments, respectively [37, 38]. In addition, participants were asked if they had even been told by a health care provider that they had a mental health disorder diagnosis (schizophrenia, depression, bipolar disorder, personality disorder). In addition to HIV PVL and CD4 cell count, other HIV care indicators included having an HIV medical provider in the community (i.e., linkage to community care) and self-reported adherence to ART 30 days prior to incarceration and during incarceration. Adherence was measured by a visual analog scale (VAS) where participants estimated the proportion of prescribed ART doses (0–100%) taken during the previous 30 days [39]. Optimal self-reported ART adherence was defined as ≥90%. Continuous viral load was categorized as < 200 (undetectable) and ≥ 200 copies/mL. CD4 cell count was categorized as ≤200 and >200 cell per uL.

### Data Analysis

For this analysis, only study participants with complete baseline data were included. The analysis was stratified by gender in order to assess differences among female, male, and transgender study participants. Depending on the data distributional properties, frequencies and percentages, or means and standard deviation (SD) were calculated for demographic characteristics, criminal justice history, sexual and substance use behaviors, healthcare utilization, and HIV care. For categorical variables, gender differences were examined by chi-square tests or Fisher’s exact tests, and for continuous variables, these differences were assessed by Student’s *t*-tests or Wilcoxon rank sum tests. Statistical significance was set at p = 0.05, and all data management and analyses were conducted using SAS 9.3 (Cary, NC).

### Institutional Review Board Approval

This study was approved by the institutional review boards of The Miriam Hospital and The George Washington University. Because this study included prisoners, the protocol was also approved by the U.S. Office of Human Subjects Research Protection. A Certificate of Confidentiality was obtained to further protect the responses of the study participants, and the study was registered with ClinicalTrials.gov (NCT01721226).

## Results

**Fig 1:**
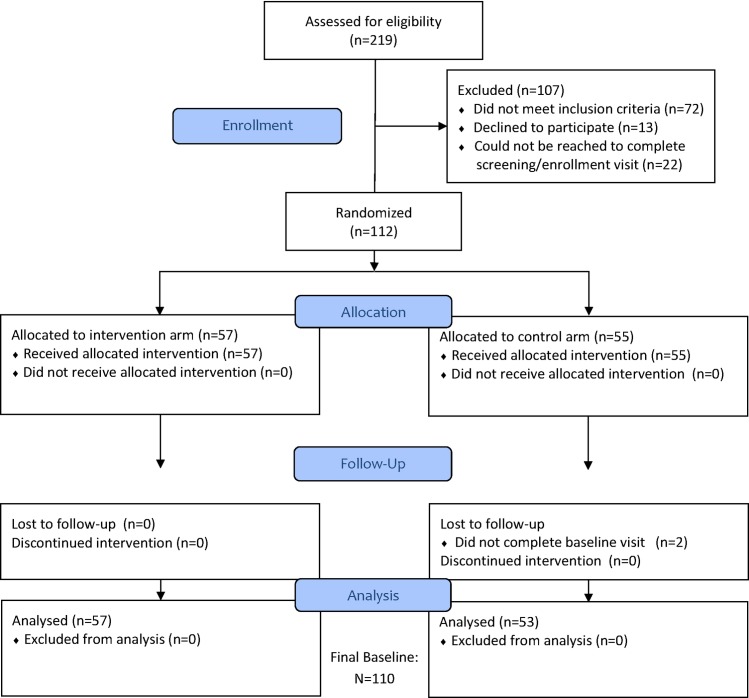
CONSORT Diagram.

A total of 497 persons were pre-screened and 219 were assessed for study eligibility. Of the 112 persons who provided informed consent and were enrolled in the study, 110 completed baseline data collection and were included in this analysis (see CONSORT flowchart in Fig 1).

Table 1 displays the demographic characteristics and site of enrollment for these participants; 70% were enrolled from the community and 30% were enrolled within the DC DOC. Mean age was 40 (SD = 10.5), and most were Black or African American (85%). The majority of participants were male (58%), 24% were female, and 18% were transgender, all of whom were MTF transgender women. There were no differences across gender groups in race and lifetime number of incarcerations (mean 10.8, SD = 9.9). The length of last incarceration was shortest for women, at 4.8 months (SD = 11.5) compared to 19.8 months (SD = 47.4) for men and 8.9 months (SD = 21.4) for transgender women (p<0.0001).

**Table 1:** Demographic Characteristics of CARE+ Corrections Study participants, stratified by gender (N = 110).

	Male		Female		Transgender (MTF)		Total		p-value
**Enrollment location n (%)**									0.1286
Community	40	(62.5)	21	(80.8)	16	(80.0)	77	(70.0)	
DC Department of Corrections	24	(37.5)	5	(19.2)	4	(20.0)	33	(30.0)	
**Race/Ethnicity n (%)**									0.7504
Black/African American	56	(87.5)	21	(80.8)	17	(85.0)	94	(85.5)	
White	1	(1.6)	2	(7.7)	1	(5.0)	4	(3.6)	
Hispanic	1	(1.6)	0	(0.0)	0	(0.0)	1	(0.9)	
Other	6	(9.4)	3	(11.5)	2	(10.0)	11	(10.0)	
**Age—Mean (SD)**	42	(10.7)	39	(9.2)	35	(9.4)	40	(10.5)	**0.0251**
**Sexual orientation n (%)**									**0.0023**
Heterosexual or straight	51	(79.7)	24	(92.3)	9	(45.0)	84	(76.4)	
Homosexual, gay or lesbian	8	(12.5)	1	(3.9)	9	(45.0)	18	(16.4)	
Bisexual	5	(7.8)	1	(3.9)	2	(10.0)	8	(7.3)	
**Length of last incarceration, months—Mean (SD)**	19.8	(47.4)	4.8	(11.5)	8.9	(21.4)	14.3	(38.3)	**< .0001**
**Number of times in jail/prison, lifetime—Mean (SD)**	10.3	(8.6)	11.8	(13.2)	10.7	(8.5)	10.8	(9.9)	0.9067

Bold text indicates p<0.05.

Table 2 displays sexual behaviors, substance use and mental health characteristics. Nearly half (47%) of the study sample had engaged in condomless sex in the three months prior to their last incarceration, but this did not differ across genders. However, engaging in exchange sex was significantly higher for women and transgender women (54% and 65%, respectively) compared to men (12%, p<0.001). Among men and transgender women, 27% of men and all transgender women reported having had sex with a man (p<0.001), and 17% of men and 79% of transgender women reported having anal sex with a man in the three months before their last incarceration (p<0.001).

**Table 2:** Sexual risk behaviors, substance use, and mental health disorders among CARE+ Corrections Study participants, stratified by gender (N = 110).

	Male		Female		Transgender (MTF)		Total		p-value
	n	(%)	n	(%)	n	(%)	n	(%)	
**Any condomless sex in 3 mo before last incarceration**									0.6443
No	34	(53.1)	12	(46.2)	12	(60.0)	58	(52.7)	
Yes	30	(46.9)	14	(53.9)	8	(40.0)	52	(47.3)	
**Ever had sex with a man (among men and transgender)**^**1**^									**< .0001**
No	47	(73.4)	0	(0.0)	0	(0.0)	47	(56.6)	
Yes	17	(26.6)	0	(0.0)	19	(100.0)	36	(43.4)	
**Had anal sex with man in 3 mo before last incarceration**^**1**^									**< .0001**
No	53	(82.8)	0	(0.0)	4	(27.1)	57	(68.7)	
Yes	11	(17.2)	0	(0.0)	15	(79.0)	26	(31.3)	
**Exchange sex in 3 mo before last incarceration**									**< .0001**
No	56	(87.5)	12	(46.2)	7	(35.0)	75	(68.2)	
Yes	8	(12.5)	14	(53.9)	13	(65.0)	35	(31.8)	
**Drug Dependence (TCU score) (12 mo before last incarceration)**									0.8070
No	29	(45.3)	10	(38.5)	8	(40.0)	47	(42.7)	
Yes	35	(54.7)	16	(61.5)	12	(60.0)	63	(57.3)	
**Ever injected drugs**									0.1832
No	51	(79.7)	24	(92.3)	19	(95.0)	94	(85.5)	
Yes	13	(20.3)	2	(7.7)	1	(5.0)	16	(14.6)	
**Non-injection drug use in 3 mo before last incarceration**^**2**^									0.1384
No	22	(37.3)	10	(43.5)	2	(13.3)	34	(35.1)	
Yes	37	(62.7)	13	(56.5)	13	(86.7)	63	(64.9)	
**Ever been in drug treatment**									**0.0117**
No	25	(39.1)	13	(50.0)	2	(10.0)	40	(36.4)	
Yes	39	(60.9)	13	(50.0)	18	(90.0)	70	(63.6)	
**Alcohol use (WHO AUDIT)**									0.2825
Low hazardous or harmful alcohol use	30	(46.9)	17	(65.4)	14	(70.0)	61	(55.5)	
Medium hazardous or harmful alcohol use	13	(20.3)	3	(11.5)	1	(5.0)	17	(15.5)	
High hazardous or harmful alcohol use	21	(32.8)	6	(23.1)	5	(25.0)	32	(29.1)	
**Depressive symptoms (CESD-10)**									0.7904
No	32	(50.0)	11	(42.3)	10	(50.0)	53	(48.2)	
Yes	32	(50.0)	15	(57.7)	10	(50.0)	57	(51.8)	
**Ever diagnosed with schizophrenia**^**3**^									0.5195
No	51	(81.0)	17	(65.4)	16	(80.0)	84	(77.1)	
Yes	12	(19.0)	9	(34.6)	4	(20.0)	25	(22.9)	
**Ever diagnosed with personality disorder**^**3**^									0.4152
No	55	(87.3)	19	(73.1)	16	(80.0)	90	(82.6)	
Yes	8	(12.7)	7	(26.9)	4	(20.0)	19	(17.4)	
**Ever diagnosed with bi-polar/manic depression**^**3**^									0.3946
No	28	(43.8)	9	(34.6)	8	(42.1)	45	(41.3)	
Yes	36	(56.3)	17	(65.4)	11	(57.9)	64	(58.7)	
**Positive screen for PTSD**									0.7444
No	37	(57.8)	13	(50.0)	12	(60.0)	62	(56.4)	
Yes	27	(42.2)	13	(50.0)	8	(40.0)	48	(43.6)	

1. N = 83.

2. Of those who reported ever using non-injection drugs (N = 97).

3. N = 109.

Bold text indicates p<0.05.

Drug dependence (57%), lifetime history of injection drug use (15%), recent non-injection drug use (65%), and hazardous alcohol consumption (45%) were all highly prevalent in this sample but did not differ across genders. However, transgender women were significantly more likely to have participated in drug treatment compared to men and women (90% versus 61% and 50%, respectively, p = 0.0117). In terms of mental health, depressive symptoms (52%) and previous diagnosis of schizophrenia (23%), personality (17%) and bipolar disorders (59%), and post-traumatic stress disorder (44%) were all highly prevalent but did not differ across genders.

Table 3 displays baseline HIV treatment and care indicators. Overall, more than 80% of study participants had a regular HIV healthcare provider prior to their last incarceration, and nearly all (91%) had ever taken HIV treatment. Only half (51%) indicated optimal HIV treatment adherence in the 3 months prior to their last incarceration, and this did not differ by gender. However, being on HIV treatment during their last incarceration was significantly lower for women compared to men and transgender women (67% among women versus 96% and 95% among men and transgender women, respectively, p = 0.001). In addition, all transgender women reported currently having a regular HIV provider in the community, versus 75% among men and 77% among women (p = 0.02). Ninety-three (88%) study participants completed phlebotomy and 13 had their medical charts reviewed for baseline HIV plasma viral load and CD4 counts. At baseline, 80% of transgender women, 69% of men, and 48% of women achieved HIV viral suppression (<200 copies/mL), although these differences were not statistically significant. Women had the highest proportion of having a CD4 count ≤200 compared to men and transgender women (5% for transgender women versus 8% for men and 17% for women), but this also was not statistically significant.

**Table 3:** HIV treatment and care indicators among CARE+ Corrections Study participants, stratified by gender (N = 110).

	Male		Female		Transgender (MTF)		Total		p-value
	n	(%)	n	(%)	n	(%)	n	(%)	
**Had regular healthcare provider pre-incarceration**									0.2065
No	14	(21.9)	4	(15.4)	1	(5.0)	19	(17.3)	
Yes	50	(78.1)	22	(84.6)	19	(95.0)	91	(82.7)	
**Ever been on HIV treatment**									0.9013
No	7	(10.9)	2	(7.7)	1	(5.0)	10	(9.1)	
Yes	57	(89.1)	24	(92.3)	19	(95.0)	100	(90.9)	
**HIV treatment adherence in 30 days before last incarceration**^**1**^									0.3403
0%-80%	23	(56.1)	6	(40.0)	6	(37.5)	35	(48.6)	
90%-100%	18	(43.9)	9	(60.0)	10	(62.5)	37	(51.4)	
**Took HIV treatment during incarceration**^**2**^									**0.0011**
No	2	(3.5)	8	(33.3)	1	(5.3)	11	(11.0)	
Yes	55	(96.5)	16	(66.7)	18	(94.7)	89	(89.0)	
**Currently have regular HIV healthcare provider**									**0.0245**
No	16	(25.0)	6	(23.1)	0	(0.0)	22	(20.0)	
Yes	48	(75.0)	20	(76.9)	20	(100.0)	88	(80.0)	
**Currently taking HIV treatment**									0.9306
No	9	(14.1)	4	(15.4)	2	(10.0)	15	(13.6)	
Yes	55	(85.9)	22	(84.6)	18	(90.0)	95	(86.4)	
**Baseline Viral Load**^**3**^									0.0614
<200 copies/mL	42	(68.9)	12	(48.0)	16	(80.0)	70	(66.0)	
≥ 200 copies/mL	19	(31.1)	13	(52.0)	4	(20.0)	36	(34.0)	
**Baseline CD4 Count**^**4**^									0.4229
≤200 cells/uL	5	(8.2)	4	(16.7)	1	(5.0)	10	(9.5)	
>200 cells/uL	56	(91.8)	20	(83.3)	19	(95.0)	95	(90.5)	

1. Of those on HIV treatment before last incarceration (N = 72).

2. Of those reporting ever on HIV treatment (N = 100).

3. N = 106.

4. N = 105.

Bold text indicates p<0.05.

## Discussion

HIV-infected persons with a history of incarceration in Washington, DC represent a vulnerable population, experiencing a high frequency of lifetime arrests as well as high prevalence of drug use, problem drinking, mental health disorders, and sexual risk behaviors. In our sample, engagement with HIV care before and after the most recent incarceration was high among the sample, but pre-incarceration ART adherence was sub-optimal and females had less access to ART during their incarceration. Baseline CD4 counts were lower and viral suppression was less frequent for women compared to males and transgender persons but these findings were not statistically significant. ART adherence remains a challenge for persons recently involved in the CJ system and needs to be the focus of public health efforts to reduce community viral load.

The majority of the study population reported having access to a regular healthcare provider and HIV treatment prior to their most recent incarceration (83% and 91%, respectively); however, only 51% reported optimal HIV treatment adherence on the VAS scale during the three months prior to their most recent incarceration. Nearly all (89%) reported receiving HIV treatment during their recent incarceration and 86% reported being on ART at the time the baseline assessment was completed in the community after release; 62% had a suppressed viral load based on objective laboratory data. This proportion of participants with viral suppression is higher than the estimate that only 30% of HIV-infected persons in the U.S. have achieved viral suppression; and more similar to recent studies that estimated viral suppression among persons in HIV care [40–42]. Collectively, these data suggest that this population in Washington, DC has reliable access to HIV providers prior to, during, and after incarceration, yet ART adherence could be improved and this would lead to higher rates of viral suppression. A recent study demonstrated that self-report of being engaged in HIV care was not always reliable when compared to clinic visit data [43]. Therefore, further research is needed to determine the causes of poor ART adherence in this population and the relative contribution of missed clinic visits. Regardless, there is a need to improve ART adherence among persons who are viremic and have concurrent risk behaviors given that they may transmit HIV to sex and drug using partners, supporting the premise that this is a high priority population for intervention.

The relatively large proportions of transgender women and men who have sex with men (MSM) that were recruited into the study was not anticipated. These groups, particularly HIV-infected MSM and transgender women, remain largely hidden within correctional populations and few studies have estimated their prevalence. The multi-site Enhance Link Initiative enrolled over 1200 HIV-infected persons with a history of jail incarceration and among these, 20% identified as MSM or bisexual [44], which was consistent with the proportion observed in our study. These findings and our results suggest that HIV-infected MSM, bisexuals, and transgender women may represent a larger proportion of HIV-infected persons behind bars than previously recognized. More research is needed to understand the epidemiology, risk profiles, and unique barriers to health services that these persons are likely to encounter in the coercive and highly stigmatized environment of correctional facilities.

The study revealed some important gender differences among our study population. Women exhibited worse outcomes compared to both men and transgender women, which is consistent with the literature [16, 17, 45, 46]. Women in our study were less likely to receive ART during their incarceration compared to men and transgender women and were least likely to have viral suppression at the baseline assessment. Being less likely to receive treatment during the recent incarceration may have been mediated by having shorter incarcerations. Women in our sample receiving ART had longer incarceration periods (3.38 months, SD = 2.14) compared to women who did not receive ART (mean = 0.74 months, SD = 2.14) (p = 0.003). Prior research has found that acceptance of ART in a correctional facility among women can be affected by stigma, perceived trust of a provider, and satisfaction of care [47], but there are few studies surrounding these issues and results are inconclusive. For example, contrary to our findings, another study found that even with shorter incarcerations, women were more likely to achieve viral suppression during incarceration compared to men [46]. More research is needed to understand acceptability of ART in a correctional setting and if shorter incarceration periods impose structural barriers (e.g., waiting to be seen by a provider) that reduce access to ART among women. This difference in access to ART is important because it is well documented that women, on average, serve shorter sentences compared to men [48].

Transgender women in our sample were more likely to have an HIV care provider prior to incarceration and at the baseline visit compared to both men and women. The reasons for these findings remain unclear, yet this is encouraging given other studies have shown HIV-infected transgender women to have poorer access to care including ART and a greater number of social and structural barriers to care [49–55]. Our findings suggest qualitative research is needed to identify both facilitators and barriers to HIV care and ART among transgender persons who have a history of incarceration.

Our study had several limitations. The generalizability of our findings to other correctional populations is limited by several factors: the sample size was small and therefore lowered the power to detect significant differences between gender groups that may have existed; the study population was relatively homogeneous in terms of race (85% were Black/ African-American); and one-half of study participants received a phone which may have prevented some persons from enrolling given there was a 50% chance of not receiving a phone. We opted to recruit study participants from the DC DOC facilities and from the community following release due to difficulty in launching recruitment inside of the DC DOC. This resulted in a more heterogeneous population that may differ with respect to HIV care and risk behaviors. However, there were few significant differences in risk behaviors by enrollment site, and no significant differences in demographic characteristics or HIV care (data not shown). Community recruitment largely occurred through social support agencies that supported returning citizens. This may have introduced some selection bias favoring HIV-infected persons who were engaged with these agencies; however, we also recruited through street-outreach in an effort to enroll a more representative population of returning citizens. Our analysis of baseline viral suppression included both laboratory testing conducted by the study and PVL data obtained from medical chart review, which increased the variability of length of time between study entry and assessment of viral suppression. Despite these limitations, this analysis represents a unique examination of HIV care and risk behaviors among recently incarcerated persons, including transgender women.

## Conclusions

Improving HIV treatment outcomes among CJ-involved persons is an important component to the broader STTR strategy given the vulnerability of this population and the fact that the vast majority of incarcerated HIV-infected persons eventually return to their communities. Our findings emphasize that improving ART adherence among CJ-involved persons remains a high priority for intervention, particularly among CJ-involved women who are at greatest risk of poor HIV outcomes. Differences in HIV care indicators and risk behaviors among men, women, and transgender women further support the need for additional research to develop gender-specific interventions to address the unique needs of each group.

## Supporting Information

S1 File IRB-approved Study Protocol. (DOCX)

S2 File Baseline CONSORT 2010 Checklist. (DOC)

## Abbreviations

CJ: criminal justice
ICT: information and communication technology
ART: antiretroviral treatment
MTF: male-to-female
STTR: Seek, Test, Treat, and Retain
DC DOC: District of Columbia Department of Corrections
MSM: men who have sex with men
