# Supplementary material for: Gender Differences in HIV Care among Criminal Justice-Involved Persons: Baseline Data from the CARE+ Corrections Study
Source: PLoS One. 2017 Jan 12;12(1):e0169078. doi: 10.1371/journal.pone.0169078 (PMC5231337; doi:10.1371/journal.pone.0169078)
Supplement: S1 File — (DOCX) [file pone.0169078.s001.docx]

**CARE+ Corrections: Technology for Jail HIV/HCV Testing, Linkage, and Care (TLC)**

**RCT protocol (without appendices)**

**v. 9-2-2014**

Principal Investigators: Curt Beckwith (Miriam Hospital), Irene Kuo (George Washington University), Ann Kurth (New York University)

Study site: Washington, DC

**Background:**

The Washington, DC Department of Corrections (DOC) Central Detention Facility (CDF) has conducted routine opt-out HIV testing since 2006. Approximately 3% of those screened for HIV were infected^1^, of which one-third were newly diagnosed infections^2^. These data strongly supports the application of Seek, Test, and Treat within jails in order to help support linkage to care among detainees with known HIV infection upon release back into the community.

For HIV-infected inmates, release into the community has been shown to be detrimental to antiretroviral therapy (ART) adherence and maintenance of HIV care^3-5^. The benefit of treating HIV-infected jail detainees is diminished if treatment cannot be continued and maintained after community re-entry. New and innovative methods including tools for real-time communication need to be developed to ensure ART adherence and linkage to care for HIV-infected jail detainees being released. To address these needs, we propose to develop information and communication technology (ICT)-based tools to facilitate the delivery of education and counseling regarding the importance of ART and HIV care adherence. If these tools are found to improve adherence to ART, enhance linkage to community care, and are found to be cost effective, they have the advantage of being readily disseminated to a broad array of correctional facilities.

**Specific aim:**

This study will investigate whether an intervention (CARE+ Corrections) delivered to HIV-infected detainees within the DC Department of Corrections (both the Central Detention Facility and the Central Treatment Facility (CTF)) and recently-released ex-detainees in the community can improve linkage to community HIV care and adherence to HIV medications after release. The intervention is modeled after **CARE**, which is a technology based HIV-counseling tool used in other settings. We have adapted CARE to create **CARE+ Corrections**, a tool designed specifically for incarcerated and recently-released populations. Counseling via CARE+ Corrections will be delivered pre-release or within 6 months after release and will consist of a one-time computerized counseling session delivered by a tablet computer that will provide interactive counseling related to risk behaviors, linkage to community HIV care, and HIV medication adherence. This counseling session will be approximately 30-40 minutes in length and will be conducted within the DOC facilities or in a community setting. CARE+ Corrections will be coupled with a cell phone/text messaging intervention that will be delivered in the community, after release from a correctional facility. The cell phone/text messaging intervention will consist of participants receiving a cell phone, or use of an existing personal cell phone, that the study team will use to deliver reminders about HIV medical appointments and taking HIV medications. The study is a randomized controlled trial where one-half of the participants will receive the intervention (CARE+ Corrections plus the community-based cell phone/text messaging intervention) either inside the correctional facility, in addition to standard discharge planning services, or in the community right after release. The other one-half of study participants will view an educational video related to the prevention of overdose following release from the correctional facility, in addition to standard discharge planning services, or in the community right after release. We will follow all participants for 6 months after release/study entry during which we will conduct follow-up assessments at 12 week and 24 week appointments to determine if the linkage to care and adherence to HIV medications were higher in the intervention arm.

**Enrollment goal:**

We plan to enroll, over a 2-year study period, up to 150 HIV-infected detainees and recently-released ex-detainees within the DC Department of Corrections facilities and in the community. Participants will be enrolled inside the DC Department of Corrections (DOC) facilities and or in other private designated space (e.g., within a local CBO or in GWU research clinic space) in the community within 6 months of release from a correctional facility.

Detainees will be recruited from the Central Detention Facility (CDF) or from the Correctional Treatment Facility (CTF), both under the jurisdiction of the DC DOC. These facilities together will be referred to from here on as “DOC”. Correctional facilities refer to jail, prison, halfway houses and other restricted settings. Recently-released ex-detainees will be recruited from local community-based organizations (CBOs) that work closely with returning citizens and venues that cater to this population (see below) (see attached letter of support from the Visitors’ Services Center: Appendix O).

**Study approval:**

Prior to study implementation, the study protocol will be approved by the George Washington University IRB (primary), the Miriam Hospital IRB, and the Office of Human Subjects Research Protection (OHRP). Following these approvals, the protocol will be reviewed and approved by the Washington, DC DOC.

**Study procedures:**

- See Appendix A for a diagram of study flow.
- Recruitment: The study will be conducted in a private space designated by the DOC Administration and Security staff or designees for detainees in DOC facilities, or in a private space (e.g., within a local CBO or in GWU research clinic space) for community-recruited individuals.

Potential participants will be HIV-infected detainees and ex-detainees. Our study will include the following recruitment methods. Potential participants will be recruited in the DC DOC facilities or through partnering CBOs or community referrals. Community participants will also be recruited from a variety of health and non-health related organizations and venues that cater to this population, including offices working with returning citizens, local establishments geared toward meeting the needs of this group, local newspaper advertisements, electronic advertisements including Craigslist.com, fliers posted at frequently-visited venues, etc, as well as through street-based recruitment. We will also contact interested individuals registered in the GWU Potential Participant Cohort (IRB #031140) for screening for this study.

- To identify potential participants inside the DC DOC facilities, the following strategy will be used. DC DOC discharge planners and case managers currently maintain a list of inmates who are scheduled to be released to the community and are slated for a medical visit, HIV discharge planning or a meeting with a case manager. The medical staff, discharge planners, case managers, and/or others working with soon-to-be released detainees will present a flier (see Appendix B: Recruitment Fliers) to inmates and refer interested individuals to meet with GWU staff in a pre-designated, private location in the DOC facilities to learn more about the study. The inmate would be screened for eligibility and consented, if willing and eligible to join the study. GWU staff will not have access to the pre-release list or names of individuals prior to the consent process.
- For individuals in the halfway houses and other correctional facilities who might be eligible, fliers may be distributed and posted (see Appendix B: Recruitment Fliers) to residents to refer those interested in the study to contact GWU staff to learn more about it.
- To identify potential participants in the community, the following strategy will be used. The GWU staff will work with partnering CBOs in the Washington DC area who operate in local correctional facilities and have direct contact with inmates, individuals who are in other correctional settings or other returning citizens in the community. (Referrals may also be made through word of mouth.) Representatives from the DOC and other correctional settings and partnering CBOs who work within the jail may offer inmates about to be released from these facilities or recently-released consumers they serve a flier (see Appendix B) about our study. They will refer interested individuals to meet with GWU staff after release in a pre-designated, private space (e.g., at local CBOs or in GW research clinic space) to learn more about the study. The ex-detainee will be screened for eligibility and consented, if willing and eligible to join the study. GWU staff will not have access to the names of individuals prior to the consent process.
  - For participants recruited inside the DC DOC facilities, a “post-release” visit (please see below) will be scheduled based on their release date. For participants enrolled in the community, follow-up visits will be scheduled based on the date of completion of the baseline visit.

***Screening and enrollment visit:***

- An interviewer-administered eligibility screener will be used to determine eligibility of potential study participants (see Appendix E: CAPI Questionnaires—eligibility screener included in baseline CAPI Questionnaires).
- Before administering the eligibility screener, a Verbal Informed Consent will be administered (see Appendix C: Screening Verbal Informed Consent Form). For recruitment conducted inside the DC DOC facilities, once the detainee has provided verbal consent, the GWU study RA will administer the eligibility screener. For recruitment conducted through partnering CBOs or community referrals, GWU staff will ask individuals to confirm their HIV status. For these individuals, GWU staff will also confirm that their release from a correctional facility occurred within the past 6 months. Once HIV positive status and date of release within the past 6 months has been confirmed and the ex-detainee has provided verbal consent, the GWU study RA will administer the eligibility screener.

Eligibility criteria include:

- Currently detained in DC DOC facility or released from the jail, prison, halfway house or other correctional facility 6 months ago or less
- Age ≥ 18
- English speaking
- Able to provide informed consent for research participation
- Anticipated release from jail, prison, halfway house or other correctional facility to the community (or living in the community if recently released ex-detainee)
- Confirmed to be HIV-infected by self-report
- Live in the metropolitan Washington, DC area.
- Able to read at 8^th^ grade level as assessed by brief literacy screen (embedded in Eligibility screener)

Exclusion criteria include:

- Expected release to a restricted setting (or currently living in a restricted setting if recruited in the community) that limits/restricts cell phone use, such as a residential drug treatment program, sober house, half-way house, or similar. If an individual is currently housed in a halfway house or other restricted setting, they may be eligible for recruitment after release from this facility.
- Detainees or recently released ex-detainees who meet the eligibility criteria will be offered the opportunity to participate in the study. Participation will be voluntary. Detainees who do not meet eligibility criteria or choose not to participate will return to their previous location as determined by the security staff. The standard of care for HIV discharge will not be affected by their refusal to participate.
- Detainees or recently released ex-detainees who elect to participate will complete written, informed consent for study participation with the study research assistant (RA). The informed consent form will be read aloud to the participant (see Appendix D: Informed Consent Forms). A copy of the informed consent will be provided to the participant. For participants recruited inside the DC DOC facilities, the GWU RA will provide the option of leaving their copy with the RA in order to protect confidentiality and receiving it at their next study visit after being released to the community. The RA will maintain a file of completed informed consents at the administrative study office at the Department of Epi/Bio at GWU in a locked file cabinet.
- Consenting study participants will also be asked to provide consent for release of medical records from the DC DOC facilities and any community medical providers that they may come into contact with during the course of the study.
- A study locator form requesting contact information for participants after release in the community will be completed. In the case of recently released ex-detainees the locator form will include participants’ current and anticipated contact information.

For community-recruited individuals, the baseline visit will occur within one study visit as much as possible. However, for individuals recruited inside correctional facilities, the baseline visit may be split across multiple time points if necessary, as determined by constraints of that facility.

***Baseline visit 1:***

- Within 1 week of the screening and enrollment visit, all study participants will return to complete a baseline assessment via computer-assisted personal interview (CAPI) (See Appendix E).

- An RA will assist with the administration of the CAPI. This will be conducted in a pre-designated space that affords confidentiality. The CAPI will be interviewer-administered by the RA who will set up the computer, ask questions to the participant and record the responses in the laptop/tablet. Participants recruited inside the DC DOC facilities will complete the “DOC Baseline CAPI” (lasting approximately 30 to 40 minutes) inside the DOC and the “Community Baseline CAPI” (lasting approximately 20-30 minutes) at the post-release visit. Participants recruited in the community will complete the “Combined Baseline CAPI” (lasting approximately 50-60 min) at enrollment.

.

- The CAPI will assess the following domains (see Appendix E for full CAPI content):
- Demographics
- Service Utilization
- Access to Care
- Mental Health
- Sexual Risk Behaviors
- Substance Use
- HCV/HBV/STI/HIV Testing & Treatment
- HIV Medication Adherence
- Post-Release Contact with HIV Healthcare Provider
- Criminal Justice
- Post-Release Concerns

Note: The baseline assessment may occur during the screening, consenting, and enrollment visit, as determined by the facility. In addition, due to operational time constraints in the facility, some of these sections may be administered during the post-release community visit.

***Baseline visit 2:***

Within 1 week of baseline visit 1, participants will complete baseline visit 2, which includes random assignment to the study arm, updates to the study locator information, and medical chart review. Depending on the operational time constraints in the facility and in the community GWU research space (some of which is rented from local CBOs), baseline visit 2 may occur immediately after the screening, consenting, and enrollment process, as determined by the facility or depending on time constraints at the GWU research clinic space.

For participants recruited in the community, a blood draw will be conducted to provide a baseline measurement of plasma viral load and CD4 cell count (baseline only) (see 12-week/24-week study visit section for description about blood draw). Study participants will be randomized at this point to either the 1) intervention arm or 2) the control arm. A 1:1 block randomization scheme will be created using a SAS program to select random assignments. Blocks of 4 will be used to ensure equal population of each group during the course of the study. The study coordinator will manage the randomization list. Prior to the visit, the RA will consult with the study coordinator for the randomized assignments. At the beginning of this visit with the participant, the RA will administer either the intervention (CARE+ Corrections tool) or the control condition to the participant based on their assignment.

- Intervention Arm:

Participants will complete the CARE+ Corrections counseling session, which will last less than 30 minutes. The CARE+ Corrections session will consist of the following:

- The CARE+ Corrections tool is a computerized, one-session motivational interview (for an outline of the CARE+ Corrections tool, see Appendix F: CARE+ Corrections Flow Chart and Excel Spreadsheet).

- The CARE+ Corrections session will be conducted using a laptop computer or tablet with a touch screen and consists of an audio-assisted session in which participants provide responses to questions about demographic characteristics, sexual risk behaviors and attitudes, substance use, mental health, and HIV treatment and adherence.
- After the computerized assessment is completed, the CARE+ Corrections tool will provide tailored feedback based on their responses to the above questions.

- The participants will have the option of selecting up to three skill-building videos on the CARE+ Corrections tool to help support the feedback they received from the initial assessment.
- The participant will work with the assistance of the CARE+ Corrections tool to develop a health promotion plan to support HIV treatment adherence and/or HIV care linkage in the community based on the feedback received from the tool.
- The CARE+ Corrections tool will review the plan steps and then the plan will be printed out.
- The participant will have the option of keeping the printout for him/herself after the visit or having the printout retained by the RA to be given to the participant at the baseline visit 3 (post-release; see below) in order to maintain confidentiality of study participation.
- The RA will be present to assist the study participant if needed and audio headphones will be provided to ensure privacy of responses. The CARE+ Corrections session will last less than 30 minutes

The RA will then review the post-release/follow-up study procedures with the study participant.

Participants recruited inside the facility will receive typical discharge planning services as conducted by the staff at the facility. For example, typical discharge planning for the DOC includes:

- 30 day supply of HIV medications (if patient is prescribed HIV medications)
- Appointment with community provider at Unity Health Care (contracted provider within the DC Jail) or referral to another community HIV provider
- DOC medical records

At this visit, a post-release/follow-up appointment will be made for all study participants. In addition, study participants will be given study materials that provide information about the study clinic location and a toll-free number to call for a reminder of the date and time of the post-release/follow-up appointment. In addition, participants recruited inside the facility will be given a flier reminding them to check in with the study staff upon release (Appendix G: GWU Marketing Materials).

- Control Arm:

Participants will view an educational video on opiate overdose (OD) prevention. Headphones will be provided for viewing. The video will be less than 25 minutes in length. Below is an outline of the information provided in the OD video (see Appendix H: Overdose Prevention Video Script, “Staying Alive on the Outside,” for the video script):

- - - Stories of overdose after release from the correctional setting: release and relapse
    - Statistics on OD upon release
    - Steps to preventing overdose
      - seeking treatment
      - make a plan to stay clean
    - Education about opiate, tolerance and purity, and danger of mixing opiates with other drugs/alcohol
    - Signs of overdose
    - How to respond to an overdose:
      - chest noogies
      - call 911
      - rescue breathing
      - Narcan
      - rescue position

The RA will then review the post-release/follow-up study procedures with the study participant, which includes collection of locator information, description of the post-release visit (see below), and explanation of continued follow-up.

Participants recruited inside the facility will receive typical discharge planning services as determined by the staff at the facility. For example, typical discharge planning for the DOC includes:

- 30 day supply of HIV medications (if patient is prescribed HIV medications)
- Appointment with community provider at Unity Health Care (contracted provider within the DC Jail) or referral to another community HIV provider
- DOC medical records

For participants in the intervention arm, recruited in the community, study staff will provide a cell phone and training on the text messaging portion of the intervention (described below).

At this visit, a post-release/follow-up appointment will be made for all study participants. In addition, study participants will be given study materials that provide information on the study clinic location, 800-number to call and make an appointment. In addition, participants recruited inside the facility will be given a flier reminding them to check in with the study staff upon release (See Appendix G).

Participants recruited in the community will receive a $50 incentive for completion of the baseline visit. Participants recruited in the correctional facility will receive their $50 incentive at the post-release follow-up visit (see below).

- Medical Chart Review: With subjects’ prior consent to the release of their medical records (see Appendix K: Medical Records Release Form) as included in the Informed Consent Form (Appendix D), relevant data will be collected from correctional facility and community provider medical records. Specific measures that will be abstracted include data from the 12 months prior to study enrollment related to HIV clinical care, comorbidities and sexually transmitted infections, and ancillary services (see Appendix I: Medical Records Abstraction Form).

The medical chart review may be conducted inside the DC DOC facility by trained GWU staff. The GWU staff member will use a CARE+ study laptop to abstract participant medical records. The laptop will be stripped down and will have no access to the internet. The study laptop will only be used to enter the information listed in the medical records abstraction form. Once the GWU staff member has entered the medical records data on the laptop, he/she will save the data on a password protected encrypted flash drive and will delete the data from the study laptop. The GWU staff member will transport the flash drive to the GWU offices and store the flash drive in a locked file cabinet within a locked closet to which only GWU study staff will have access.

If medical records are available from correctional facilities and/or community clinics, the medical records will be obtained for data abstraction at GWU.

- Tracking of release dates: Anticipated release date (only for participants recruited inside the correctional facility), sentences and charges, dispositions, incarceration periods and facilities, release statuses, discharge planning and case management services, post-release residential programs, and community health referrals will be provided by staff at the correctional facilities and confirmed during the DC DOC record chart review (see Appendix J: DC DOC Abstraction Form) based on subjects’ prior consent to the release of their DC DOC records (see Appendix L: DC DOC Records Release Form). On a regular basis, study staff will consult with staff at the correctional facilities to confirm the release of study participants. After learning about a participant’s release, the study staff member will begin to attempt to schedule the post-release baseline visit 3 within a two-week period (see Baseline Visit 3—post-release visit).

***Post-release visit:***

After learning about a participant’s release, study staff will contact the study participant using previously collected locator information to confirm or arrange for a post-release visit within 14 days after release. For community-recruited individuals, this visit will take place during the initial baseline visit when possible. An appointment will be made for the participant to meet with study staff at designated, private space (e.g., at local CBOs or GW research clinic space). At this time, the study team will update the participant’s locator information, provide a resource/referral list for medical services, and provide a $50 incentive for their time. The payment is in the form of an American Express Gift Cheque. For those in the intervention arm, study staff will provide a cell phone and training on the text messaging portion of the intervention (described below). All study participants will be reminded about the monthly check-in calls and the 12-week and 24 week visits, and an appointment will be made for their next study visit. A blood draw will be conducted to provide a baseline measurement of plasma viral load and CD4 count (see 12-week/24-week study visit section for description about blood draw).

**Intervention arm:** Participants randomized to the intervention arm will receive a cell phone/SMS texting intervention that will begin at baseline visit 3. This intervention will consist of using cell phone-delivered automated SMS text-messaging with the intent of improving linkage to community HIV care and adherence to ART (if prescribed); and reinforcing the prevention plan from the CARE+ Corrections session completed in jail prior to release. In addition to SMS text-messaging, the cell phones will provide participants with a method for contacting their HIV clinic and other services providers (AIDS service organization case manager, social workers, re-entry service providers, etc.).

The study will provide basic cell phones and pre-paid cell phone plans to study participants who do not possess a cell phone at the time of the post-release visit. For participants who do own a cell phone with text messaging capability, participants will have the option of using their own cell phones and the study will provide a standardized monetary reimbursement for the costs incurred through study activities. Participants who possess a cell phone that does not have text-messaging capability will be provided a study phone.

Provision of cell-phones: Participants who do not possess a cell-phone will be provided with a Droid 4 Prepaid Phone ($49.99), or similar phone, with an unlimited text bundle and 400 minute calling plan or similar plan (~$40/month). The cost of the phone, the initial activation fee and costs for the unlimited text and calling minutes bundle will be covered by the study. Study staff will pre-purchase the cell phones and cell phone plans. All cell phone bills will be sent directly to study staff. The text-messaging and calling plans will be renewed on a monthly basis during the study follow-up period of 24 weeks. At the completion of the follow-up period, cell phone plans will be cancelled.

Use of existing cell phone: Participants who possess a cell-phone with text-messaging capability at the time of the post-release/follow-up study visit will have the option of using their own phone to receive the SMS text-messaging intervention and to use for communication with their community providers. Participants using their own cell-phones will be asked to provide information regarding their cell phone make/model, cell phone number, and cell phone provider/plan (including text messaging and call plans). Participants who use their existing phone will be reimbursed a standardized monetary amount per month based upon the anticipated cost related to receiving study text messages delivered through the intervention, plus reimbursement for an estimated volume of calls between the participant, study staff, and community providers. Reimbursement will accrue on a monthly basis and participants will collect this reimbursement at the 12 and 24 week study visits or have the option of picking it up on a monthly basis at the GWU community research site.

Training on cell-phone intervention: All participants will receive training delivered by study staff on the cell phone intervention. This will include training on the use of the cell phone as well as how to view and respond to text messages. The staff member and the participant will practice sending and viewing text messages to confirm that the participant understands how to use the phone and text messaging. The staff member will review the text-messaging plans and the use of customized messages (as described below). To facilitate cell phone communication with community providers, the staff member will assist the participant with programming important phone numbers into the cell-phone for easy retrieval and use. Phone numbers will be obtained from the DC Ex-Offenders Returning Citizens Referral List (see Appendix M) and will include phone numbers for medical providers, community-based organizations, re-entry services, etc. The staff member will also review the lost cell phone policy (described below).

Cell-phone/SMS text messaging intervention content: Separate text messaging content will be used for linkage to care, ART adherence, and the promotion of the CARE+ Corrections prevention plan. Text-messages will be delivered through a contracted SMS provider. Participants will be asked to send a response to the text message within 24 hours when a study text message is received. For each content category, the participant will choose from pre-scripted messages or, if technically feasible, the participant will have the option of creating a customized message as described below. In addition, for each content category, the participant will be able to choose from several different text-message frequency options (such as daily, every other day, three times weekly, weekly). The frequency of text messages in each content category can subsequently be changed by the participant at the monthly check-ins according to preference. To maintain confidentiality, text messages will not contain participant names, mention of HIV infection or HIV medications, or specific providers that only provide HIV care.

Linkage to care content: Text messages will be sent to remind the participant about his/her upcoming HIV appointment in the community. In addition, the message will ask the participant to contact their provider if s/he anticipates difficulty in attending the scheduled appointment. We will ascertain the participant’s next appointment during the monthly check-in phone call in order to set up or adjust the reminder text message. The participant will choose from pre-scripted appointment reminders or, if technically feasible, will create a customized message. The linkage message chosen by the participant will be recorded by the staff member.

- Pre-scripted text message example: “Don’t forget your upcoming Unity appointment. Call the clinic at 202-XXX-XXXX if you can’t make it”.
- Customized text message example: “Don’t forget your upcoming meeting at Church. Call the pastor if you can’t make it”.

Message reminders will be sent out at pre-determined intervals (e.g., weekly in the month before the appointment and then more frequently during the week of the appointment). Changes to text message content (pre-scripted or customized) will be recorded by the RA and will be entered into the SMS database.

A query message will be sent the day after the scheduled appointment and will ask “How did your appointment go? If OK, reply 1. If not OK, reply 2.” If the participant replies “1”, no further action will be taken. If the participant replies “2”, or if the participant does not respond to the query message within 24 hours, a study staff member will call the participant on their cell phone to assess barriers to care and the staff member will provide referral information (clinic, social work, case management) as needed. Responses to these texts and action taken by study staff will be recorded by the RA and then entered into the SMS database.

Adherence to ART content: For participants prescribed ART at the time of the baseline visit 3, text message reminders will be sent to remind the participant to take his/her ART. The participant will choose from pre-scripted ART medication reminders or, if technically feasible, will create a customized message. The ART adherence message chosen by the participant will be recorded by the staff member.

- Pre-scripted text message example: “Don’t forget your medications today. They are important!”
- Customized text message example: “Don’t forget to eat skittles today. They are important!”

Participants will select from different frequency options for ART medication reminders. The preferred frequency will be recorded and will be entered into the SMS database. Participants will have the option of changing this frequency at the monthly check-in call. Changes to text message frequency will be recorded by the RA and will be entered into the SMS database. Participants in the intervention arm who are not prescribed ART at the time of the baseline visit 3 will be asked at the monthly check-in calls if ART has been started. For participants who start ART during the follow-up period, they will have the option of adding ART reminders to their ongoing intervention according to the procedures described above.

CARE+ Corrections prevention plan content: During the CARE+ Corrections session completed in jail, participants will receive counseling in a number of areas including: post-release linkage to HIV care; post-release ART adherence; sexual risk reduction counseling; substance use risk reduction counseling; and working with your HIV provider. At the baseline visit 3, a staff member will review the individual prevention plan created during the CARE+ Corrections session with the participant and the participant will decide upon a prevention message to be used in the text-messaging intervention. The participant will choose from pre-scripted prevention messages created from the CARE+ Corrections content or, if technically feasible, will create a customized message. The prevention message chosen by the participant will be recorded by the staff member.

- Pre-scripted text message examples:
  - “Don’t forget to use protection. Protect yourself and your partner!”
  - “Don’t forget to meet with your case manager. She can help!”
  - “Watch out for partying. It can make you lose focus!”
- Customized text message examples:
  - “Stay away from drinking alcohol. Makes you forget your meds”
  - “ Call your sponsor, she can help you stay on track”
  - “Talk to your partner about staying safe. That’s important for both of you”

Participants will select from different frequency options for the CARE+ Corrections counseling reminders. The preferred frequency will be recorded and will be entered into the SMS database. Participants will have the option of changing this frequency at the monthly check-in call. Changes to text message frequency will be recorded by the RA and will be entered into the SMS database.

Barriers to community care content: The CARE+ Corrections session will also identify potential barriers to community care. The participant will choose from pre-scripted prevention messages created from the CARE+ Corrections content or, if technically feasible, will create a customized message. The prevention message chosen by the participant will be recorded by the staff member.

- Pre-scripted text message examples:
- “Get your benefits/insurance programs set up: call xxx-xxx-xxxx”
- Customized text message examples:
  - “Don’t forget to sign up for that program: call xxx-xxx-xxxx”

This message will be delivered once weekly to participants during the time period between the start of the SMS intervention and the first monthly check-in (approximately 4 weeks). If a participant is re-incarcerated and released during the study follow-up period, this content area will be repeated during the 4 weeks after release.

Administrative messages to all participants:

- A welcome message during the first week after registration
- Messages reminding participants about their monthly check-in with study staff

Tracking of text messages: For each participant, a staff member will record the message reminders selected for each content category and these will be entered into the SMS database. Text messages will be automatically delivered according to the scheduled frequency. For each participant, the SMS contractor will track messages sent and received by the participant and responses sent by participant back to study staff.

Lost cell-phone policy: If a participant who receives a study phone loses the phone, one replacement cell phone/plan will be provided per participant during the 24 week follow-up period. The cell phone plan of the original phone will be terminated immediately upon learning that the phone was lost. If the second phone is lost during the follow-up period, the cell phone plan will be terminated immediately upon learning that the phone was lost and no further phones will be provided to the participant.

**Follow-up assessments**

As part of the retention strategy, study staff will contact participants by mail, phone, email, and social media messages to remind them of upcoming appointments or to reschedule missed appointments. Participants will be informed during the consent process that we may visit them at their home, work or other location where they may be found if we are unable to contact them by phone or mail. Study staff will use contact information provided by the participants to get in touch with them.

***Monthly contact by phone/in-person***

1. To enhance retention in the study, study staff will conduct monthly check-in phone calls or visits with all study participants to update locator and contact information and provide an opportunity for individuals in the texting intervention group to customize the frequency and content of their text message for the next month (including reminders for the next medical appointment, ART dosing, and prevention plan goal). In addition, participants will be asked about HIV medication adherence for that past month (See Appendix N: Monthly call Script and Medication Adherence Tool) to help validate self-reported adherence at the 12-week and 24-week in-person assessments. Study staff will also remind study participants about the next monthly call date and next study appointment date. A $10 incentive in the form of an American Express Gift Cheque will be provided for each successful monthly contact, and participants can opt to receive the incentive at that visit (if in person), arrange for another date/time for pick-up (if by phone), or receive their bundled incentives at the next study visit.

***Study Visit 2 (12-week in-person assessment) and Study Visit 3 (24 week in-person assessment) (+/- 8 weeks):***

1. The study team will confirm and update locator information and will obtain updated medical release forms at Study Visits 2 and 3 from all participants to access medical records from community clinics and correctional facility at which the participant has received care during the follow-up period (See Appendix K: Medical Records Release Forms). Specific measures that will be abstracted from the medical records include data from the follow-up period (up to 32 weeks) related to HIV clinical care, comorbidities and sexually transmitted infections, and ancillary services.
2. Participants will self-report linkage to HIV care (date(s) and location(s)) and complete a follow-up CAPI.
3. A trained phlebotomist will conduct an intravenous blood draw in which up to 20ml of blood will be drawn for plasma viral load (PVL) testing, CD4 cell count (at baseline only) and back-up specimen storage. ART resistance and tests for other related co-morbidities may be conducted on stored specimen if funding is available. The specimen will be labeled with a study ID number only.
   - - - 1. The blood specimens for plasma viral load will be processed at the GWU Jordan virology lab and spun down twice at 1600 x g for 15 minutes, aliquoted in 2.2 ml aliquots (minimum volume) and frozen at -70 to -80C within 6 hours of collection. Specimens will be labeled using freezer labels with study ID, date, study name, visit number, and specimen type (EDT PL). All specimens will be banked at the Jordan lab at GWU until required for shipping and/or testing.
         2. Specimens for viral load testing will be shipped on dry ice to Miriam Hospital bi-weekly where they will be evaluated using PCR. Viral load reports using only study ID will be sent to study staff at Miriam Hospital, who will in turn send the results to GWU study staff via secure server and/or FedEx.
         3. Specimens for CD4 cell count will be processed and sent to a local lab for testing. The specimen will only be labeled with a study ID and results will be sent to study staff via a secure web portal and/or FedEx.
         4. The results of these tests will be shared with study participants upon request, and study participants may share the results with their health clinics or medical providers if they wish to do so.
4. At the end of the study visit the staff will provide a list of referrals, a $50 incentive for completion of the visit, $10 incentives for every successful monthly phone call since the previous study visit, and a voucher for transportation. The incentives will be in the form of American Express Gift Cheques.
5. After the study visit, the study staff will confirm the participant’s engagement in care by contacting his/her medical provider and obtaining data on appointments, plasma viral load, CD4 data if available and antiretroviral medication prescriptions, given approved medical release from the participant.

**Study Termination :**

Study participants may withdraw their consent to participate in this study at any time and for any reason. The site investigator may voluntarily withdraw from the study for any reason at any time. The site investigator may also withdraw participants from the study in order to protect their safety, or the safety of site staff and/or if they are unwilling or unable to comply with required study procedures after consultation with the co-PIs and Biostatistician. Participants also may be withdrawn if the study sponsors, government or regulatory authorities terminate the study prior to its planned end date. Participants will be informed in the consent process that the investigator or the study sponsor may terminate their participation in the study at any time without their consent for any of the reasons listed above. The study staff will explain the reason for terminating participants’ involvement in the study and what arrangements will be made for continued care.

The study staff will make every effort to enroll, follow, and obtain samples from all enrolled participants according to the study procedures listed above, while recognizing that each participant may not be able to return for all visits or undergo all protocol-dictated procedures.

Every reasonable effort will be made to complete a final visit with participants who terminate from the study prior to the end of the study period, and study staff will record the reason(s) for all withdrawals from the study in participants’ study records.

**Re-incarceration Plan:**

Correctional facility rosters will be reviewed to track re-incarceration throughout the study period, allowing the study team to locate participants who are re-incarcerated as well as determine time individual participants spend incarcerated during the follow-up period.  Should a participant be re-incarcerated or put under supervision within a correctional facility at the time a 12 or 24 week assessment visit (Study Visits 2 or 3) is due, the assessment visit will be conducted within the correctional facility if possible.  The following exceptions will be made to the standard follow-up assessment visits for those conducted within the correctional setting:

1) Blood will not be drawn for plasma vial load assessment.  Instead, the correctional facility medical record will be reviewed for a HIV PVL result within two weeks of the assessment visit.

2) No incentive or transportation vouchers will be provided for completing assessment visits while incarcerated.  The participant will have the option of picking up the $50 incentive (in the form of an American Express Gift Cheque) at the GWU study office if released prior to the completion of the funding period.

**Data Transfer and Confidentiality**

***Participant Identification***

To identify potential participants, DC DOC medical staff, discharge planners and case managers will refer HIV positive inmates to meet with GWU staff. Staff members from CBOs will also refer HIV positive ex-inmates to meet with GWU staff. Potential participants will be assigned a pre-screening ID. We will also contact interested individuals registered in the GWU Potential Participant Cohort (IRB #031140) for screening for this study. An electronic spreadsheet linking participant names and pre-screening IDs will be saved on an encrypted jump drive specifically designated to house this file and will be physically secured at the GWU Department of Epidemiology and Biostatistics offices for the duration of data collection period.

Once inmates/ex-inmates, provide consent for being screened for eligibility, are screened for eligibility, and provide consent to participate in the study, GWU research staff will enroll them in the study and assign them a unique study ID number. Study IDs will be created using alphanumeric characters and will not contain any identifying information. With the exception of locator forms and informed consent forms, these study IDs will be used to uniquely identify all study documents pertaining to a particular participant. An electronic spreadsheet linking participant names and study IDs will be saved on an encrypted jump drive specifically designated to house this file and will be physically secured at the GWU Department of Epidemiology and Biostatistics offices for the duration of data collection period. The jump drive will be stored in a locked cabinet or drawer separate from the participant files (ID numbers) and consent/locator forms (named files). Only investigators directly involved in the study at GWU will have access to the identifying information. The link file will be destroyed post-study.

***Data Collection within DC DOC Facilities and GWU research clinic space***

Participant recruitment, screening, consent, and baseline CAPI administration will be performed by trained study staff within correctional facilities or a designated, private space (e.g., at local CBOs or GW research clinic space). Given the sensitive and personal nature of the information being requested, these activities will occur in a private space or other areas in the correctional facilities and GWU research clinic space that will optimize participant confidentiality. Data collection instruments, including the study laptop computer, password-protected tablet computer, and all paper documents, will be maintained and secured by the research staff while within the facilities/ GWU research clinic space. At the end of each data collection period, all study materials will be removed from the correctional facilities or GWU research clinic space by the research staff and secured in locked file cabinets at the GWU Department of Epidemiology and Biostatistics offices.

***Post-Release Data Collection***

The initial post-release visit and follow-up Visits 2 and 3 at weeks 12 and 24 will be conducted in other private designated space (e.g., within a local CBO, such as VSC or FMCS, or in GWU research clinic space). GWU research clinic space at VSC and FMCS offices are kept locked and code-entry is required to access the research space. All data collection activities in the community, including CAPIs, locator form updating, and the collection of laboratory specimens, will occur in private rooms within the VSC/FMCS clinic spaces to ensure participant confidentiality. Data collection instruments, including the study laptop computer, the password-protected tablet computer, and all paper documents, will be secured by the research staff while on site at GWU research clinic spaces and will be secured at the GWU Department of Epidemiology and Biostatistics offices when not in use.

Information gathered during monthly check-in contact will initially be recorded on paper documents secured by the research staff. These documents will be kept in locked file cabinets at the GWU Department of Epidemiology and Biostatistics offices when not in use.

***Data Management***

GWU will maintain a secure server network drive to which only designated GWU investigators will have access. The following electronic databases will be secured on the network drive:

1. QDS^TM^ warehouse for CAPI surveys
2. Test results from testing of biological specimens
3. Monthly check-in call database
4. Clinic appointment adherence assessment database
5. Merged database (with data from #1-4)
6. Medical chart review data

*Computer-assisted Personal Interview (CAPI):*

To ensure consistency throughout the study period, GWU will develop a QDS^TM^ (Questionnaire Development System) control file with specifications for the CAPI data. QDS^TM^ is software used to program survey development, collect data, and manage data collection. At the end of each day, CAPI interview files will be uploaded into the QDS warehouse via an encrypted jump drive and subsequently deleted from both the laptop computers and jump drive. Interview files and the data warehouse are password-protected.

*Data Entry:*

With the exception of locator forms and informed consent forms, data that is initially recorded onto paper documents will be directly entered into the appropriate database on a weekly basis at the GWU Department of Epidemiology and Biostatistics offices.

*Merged Database:*

At the end of each week, the data manager will combine the above-mentioned databases into a single database. Study IDs will be used to merge the databases.

***Data Progress Reports***

The data manager will produce regular reports that contain de-identified information and summarize study activity progress. Regular data reports will be presented to GWU research staff involved in data collection activities and to Principal Investigators at GWU, Miriam Hospital, Brown University, and NYU as needed.

***Monthly Data Transfer to RI CFAR***

Data De-Identification:

Identifying information that enables the investigators to readily ascertain the identity of a study participant will be replaced with a study ID. The study ID will be a combination of numbers and letters, which will have no specific meaning pertaining to participant’s private information. The participant’s private information and the key that enables linkage of the study ID to private information will be maintained separately at the GWU study office in a locked file cabinet, which (a) will have restricted access only to the investigators at GWU under the GWU IRB policies and (b) will not be released under any circumstances to Dr Tao Liu and study staff at Brown University Center for Statistical Sciences.

Data Transfer:

De-identified data will be transferred directly from GWU to Dr. Tao Liu at the Brown University Center for Statistical Sciences via a SecureTransport shared folder, to which only Dr. Liu and GWU study staff directly involved in the data transfer will be assigned password-protected user accounts. SecureTransport is a product used by the George Washington University for secure file transfer. It uses Hypertext Transfer Protocol Secure (HTTPS) and user authentication for secure communication across the internet. It has been certified Federal Information Protection Standard (FIPS) 140-2 Level 1 by the US National Institute of Standards and Technology (NIST). The secure folder will not be used for permanent data storage. De-identified data will be uploaded by GWU study staff and will remain in the folder for a maximum of 72 hours, during which time Dr. Liu will download the files before deleting them from the folder. The downloaded data will be stored on secure servers at the Brown CSS, and access will be restricted to study staff.

***Data Analysis & Presentation of Results***

Participants will be identified by their designated study IDs during all data analyses. Individual results will not be shared, and only aggregate results will be disclosed at study completion. Summary statistical data stratified by age, race or other categories may be released. An asterisk (*) will represent all cells with 3 or fewer observations. However, if the total information will allow identification of the exact person(s) represented by the asterisk, the information will not be released.

***Subject Confidentiality***

All research staff will sign confidentiality agreements prior to interacting with any study participants. All study procedures will be conducted in private, and every effort will be made to protect confidentiality and participants’ privacy to the extent possible. Also, study staff, correctional facility and CBO representatives will be encouraged to identify potential confidentiality issues and strategies to address them.

All study-related information will be stored securely at the GWU study office. All participant information will be stored in locked file cabinets and/or in lockable areas with access limited to GWU research staff. All laboratory specimens; reports; and data collection, process, and administrative forms will be identified only by a coded number to maintain participant confidentiality. All records that contain names or other personal identifiers, such as locator forms and informed consent forms, will be stored separately from study records identified by code number. All local databases will be secured with password-protected access systems. Forms, lists, logbooks, appointment books, and any other listings that link participant ID numbers to other identifying information will be stored in a separate, locked file in an area with limited access. Some members of the GWU research staff will work with participants outside of the DC DOC, the CBOs, or VSC/FMCS offices, but will follow strict procedures to limit the confidential information taken into the field and to safeguard any information collected outside of the site.

*Certificate of Confidentiality*

A Certificate of Confidentiality from the US Department of Health and Human Services will be obtained for the study. This Certificate protects study staff from being compelled to disclose study-related information by any US Federal, State or local civil, criminal, administrative, legislative, or other body.

All protected health information (PHI) will be protected according to the provisions of the HIPAA and will only be used or disclosed as allowed by the privacy rule pursuant to relevant waivers or authorizations, or as required by federal law.

***Withdrawal from the Study***

A subject may withdraw from this study at any time without prejudice. However, information collected prior to withdrawal may still be used after withdrawal.

***Study Files and Record Retention***

Link files and locator forms will be destroyed immediately post-study. Investigators will retain all additional study records at least three years, after which they will be destroyed.

**Analysis Plan**

*Summary analysis:*

Demographic characteristics (including age, gender, ethnicity, etc.) and history of prior incarceration, sexual behaviors, drug and alcohol use, and medical history (including HIV testing history, HIV/HCV status) will be summarized using means/standard deviations, medians/ranges or counts/frequencies with corresponding 95% confidence intervals (CIs) as appropriate. The summaries will be stratified by arm. An intent-to-treat analysis will be used. Baseline characteristics will be compared between control arm and corresponding comparison arm using Pearson’s Χ^2^ test for categorical variables, student’s t-test for normally distributed or Wilcoxon’s test for non-normally distributed continuous variables to assess the success of the randomization procedure. Imbalances between the study arms will be documented and investigated. Imbalanced covariates will be adjusted for subsequently through regression analysis or weighted analyses. Lost-to-follow-up (LTF) and incomplete response will also be summarized by contingency tables for each arm stratified by gender and study site, and will be compared using Pearson’s Χ^2^ test to examine whether selection bias is induced by LTF. A diagram of LTF status will be created to summarize the disposition of participants at each study visit.

*Analysis of primary and secondary outcomes:*

The longitudinal nature of the study (containing a baseline assessment before intervention and two follow-up visits after jail release) will result in repeated assessments of primary outcomes (plasma viral load suppression at Visits 2 and 3) and secondary outcomes (e.g., proportion linking to community HIV care at 30 days, 60 days, 90 days, etc.). These outcomes will be analyzed in the unified framework of generalized linear models (GLMs) with appropriate link functions (logit for dichotomized outcomes, e.g. linkage to care, detectable PVL; identify link for continuous outcomes, e.g. log-transformed PVL; or log link for count outcomes, e.g. # of non-main sexual partners). The setup of GLMs involves relating the expected outcome to a linear function of explanatory covariates, where the explanatory covariates will include a dummy variable encoding the treatment (=1) versus control (=0), dummies for study site and gender, relevant demographic covariates, and their interactions. Depending on the analysis objectives, three GLM model structures will potentially be used: 1) *Marginal models* will be used to analyze population-level intervention effect. Within-individual correlation of repeated measures will be accounted for using Generalized Estimating Equations (GEE),^6,7^ which yields consistent parameter estimates and robust standard error estimates. 2) *Random-effect generalized linear models* will be used to estimate the intervention effect at the individual-level.^8^ For those outcomes with only one follow-up visit, the model will be estimated using conditional logistic regression for binary outcomes or difference-on-difference regression for continuous outcomes for better estimation efficiencies. 3) *Markov transition models* will be used to investigate the intervention effect on the individual behavior change as previously conducted by this group,^9^ and includes the prior history of the outcomes as regression covariates; therefore, the transition model emphasizes more the effect of the intervention on outcome changes given the prior value of the outcome. Using these model setups, we will examine the different aspects of intervention for SA2 and SA3. These models will also allow us to quantify the differences between the two study sites and differences between male and female by testing the interaction between study arm and site, and potentially the interaction between study arm and gender.

*Strategy of handling missing data:* Differential LTF between arms could eliminate the randomization effect. The first round of analyses will make the “missing at random” (MAR) assumption, which states that after conditional on baseline covariates, there is no systematic difference between those LTF and those retained in the study. With the MAR assumption, we will construct logistic regression models that express “missingness” as a function of covariates, and use the logistic model to calculate the propensity score for “missingness” for each individual. We will use the propensity score in terms of inverse probability weighting to adjust for the selection bias due to missing data in our regression analysis.^10^ Afterwards, we will relax the MAR assumption by conducting a sensitivity analysis through considering a family of plausible “not missing at random” models using the techniques outlined by Diggle and Kenward^11^ and Daniels et al.^12^

**Study Timeline:**

Up to 150 participants will be enrolled in the study. Recruitment will begin in August 2013 for 18-24 months, and follow-up visits will conclude in August 2015. Data analysis and dissemination will commence soon after the first follow-up visit and will continue through to the end of the study (June 2015).

| **Activity** | **Time frame** |
| --- | --- |
| IRB submission | June 2012 |
| IRB approval | September 2012 |
| Recruitment | August 2013 – February 2015 |
| Follow-up visits (12 and 24 weeks) | November 2013 – August 2015 |
| Data analysis and dissemination | August 2013 – June 2016 (with no cost-extension) |

**Appendices:**

1. Study Flow Diagram
2. Recruitment Fliers
3. Screening Verbal Informed Consent Form
4. Informed Consent Forms
5. CAPI questionnaires
6. CARE+ Corrections Flow Chart and Excel Spreadsheet
7. GWU Marketing Materials
8. Overdose Prevention Video Script, “Staying Alive on the Outside”
9. Medical Records Abstraction Form
10. DC DOC Abstraction Form
11. Medical Records Release Form
12. DC DOC Records Release Form
13. Returning Citizens Referral List
14. Monthly call Script and Medication Adherence Tool
15. Letter of support from the Visitors’ Services Center
16. Locator Form
17. Appointment Confirmation Forms (Baseline, 12-week follow-up, 24-week follow-up)
